# Epidemiology in conflict – A call to arms

**DOI:** 10.1186/1742-7622-1-5

**Published:** 2004-10-15

**Authors:** Clarence C Tam, Ben A Lopman, Olga Bornemisza, Egbert Sondorp

**Affiliations:** 1 Infectious Disease Epidemiology Unit, Department of Infectious & Tropical Diseases, London School of Hygiene & Tropical Medicine, London, UK; 2 Environmental and Enteric Diseases Department, HPA Communicable Disease Surveillance Centre, London, UK; 3 Department of Infectious Disease Epidemiology, Imperial College London, London UK; 4 Health Policy Unit, Department of Public Health Policy, London School of Hygiene & Tropical Medicine, London, UK

## Abstract

In this first special theme issue, *Emerging Themes in Epidemiology *publishes a collection of articles on the theme of *Epidemiology in conflict*. Violent conflict is an issue of great sensitivity within public health, but more structured research and reasoned discussion will allow us to better mitigate the public health impacts of war, and place the public health community in a more informed position in discussions about possible interventions in future conflicts.

*"And there went out another horse that was red: and power was given to him that sat thereon to take peace from the earth, and that they should kill one another: and there was given unto him a great sword." *– Revelations 6:4

Perhaps more than any other previous conflict, the recent war in Iraq has stirred the public health community, with numerous pages in general medical and scientific journals devoted to contributions condemning the basis of the war, condemning those condemning the war and condemning editors for delving into politics by publishing these condemnations [1]. For all this self-castigation, however, the public health message was notably absent from mainstream media and political discussion.

The issue of war is particularly sensitive in the field of public health, some might even say taboo. It is not seen as appropriate to openly denounce war. Perhaps we feel that becoming involved in what is largely perceived to be a political issue constitutes a threat to that most precious of our ideals, that of objectivity. Or maybe we are uncomfortable with the thought of being associated with military activities. Specifically, the opportunistic use of military language in public health issues has not been particularly welcome. Political calls for war on the societal problems of drugs and cancer have been accused of victimizing individuals and setting implausible goals for their prevention and control [2-6]. Ironically, we have yet to publicly declare war on the greatest of societal ills, war itself. The dilemma facing a scientific editor attempting to circumnavigate the turbulent waters of politics is eloquently summarized in a recent *British Medical Journal *editorial: to do nothing is as much a political decision as to challenge an issue head-on [7].

Given such an environment, readers may find it surprising that we should devote our first special issue to the subject of *Epidemiology in conflict*. Our reason for doing so is simple: regardless of the political context, war is bad for your health. The politicization of war within public health is unfortunate; re-framing public health questions within a political context prevents us from conducting informed discussion and finding rational solutions to them. Here, we draw a parallel with the early efforts to communicate the link between smoking and lung cancer. In his reflections on the subject [8], Ernst Wynder describes the opposition he encountered when trying to relay the findings from the first studies establishing the epidemiologic link. Opposition came not just from the conflicted interests of governments, the media and tobacco companies, but also from within the public health profession, which was at the time dominated by physicians, many of whom were smokers, were unused to the interpretation of epidemiologic data or simply thought the association to be implausible. Smoking was (and continues to be) a highly political issue, yet few would argue in retrospect that Doll, Hill, Wynder and the early proponents of the smoking-lung cancer association should, in the face of incontrovertible evidence, have done anything else than to publicize this link. Here the parallel ends, however. Given adequate knowledge about the risks to their health, an individual may freely choose to smoke and accept responsibility for any ensuing personal health consequences. Equally, they may choose to avoid these risks by not smoking altogether (although we stipulate that the effect of passive smoking is contentious here). An individual cannot choose not to go to war or have war inflicted upon them. Such decisions are carried out by governments, insurgents or other groups, many of which are not accountable to individuals. In this scenario, the role of academics and professionals, as well as professional associations and non-governmental organizations in informing governments and the public and raising issues that affect society as a whole becomes even more important. The duty of health professionals as advocates for public health is emphasized by Wynder and is equally applicable today and perhaps even more so to the issue of war:

"*...the consensus of opinion among experts is not sufficient to create action unless such consensus is translated into preventive or control measures.... Scientists and physicians cannot be content with discoveries until their beneficial or protective outcome for the population has been fully realized. This means that the members of the scientific and medical community must become more proactive in public health matters *[8]."

The issue of consensus is important, but difficult. Some will argue that war is inherently bad for public health and should always be opposed. Others will consider some wars justifiable if they address gross injustice or human rights abuses. And yet others, in line with the Geneva Conventions, will see wars, just or not, as inevitable and will want to focus on mitigation of human suffering.

Regardless of one's viewpoint, each of these positions needs to be supported by an evidence base with answers to the questions of when, how and why war is bad for public health, as well as how the adverse health effects of war may be prevented. Therein lies the greatest challenge for epidemiologists. Conflict situations deny us access to data and dissolve the health infrastructures on which we rely for the collection of epidemiologic information. In the face of such challenges, the authors of the articles in this special issue deal with a broad range of issues of great relevance to epidemiologists.

Mock *et al*. [9] argue that the interface of HIV/AIDS and conflict is more complex than is usually assumed. It is often said that war exacerbates the HIV epidemic, but the ecologic evidence suggests that this is not always the case. The authors examine the complexities of this issue and analyze how conflict can both exacerbate and retard the spread of HIV.

McDonnell *et al*. [10] evaluate the role of epidemiologists in conflict settings. Present barriers to effective engagement stem from the fact that epidemiologists do not receive training on issues pertinent to their operating in conflict-affected areas. Perhaps most important are appropriate communication skills to enable epidemiologists to present their message clearly as health related rather than political.

Roberts and Hofmann [11] place the work of humanitarian agencies under the epidemiologist's gaze. All too often such agencies, with the best intentions, measure success in terms of process – how many meals were handed out, how many vaccinations were given? From a health perspective this is only part of the story. Did these actions really have a positive impact on health? The difficulties of collecting such information mean that humanitarian interventions rarely incorporate tangible, impact-driven outcomes as priorities. This article proposes a framework for assessing the impact of aid on health.

These papers provide a foundation on which we hope authors will continue to build so that a comprehensive range of relevant topics on this subject may be compiled. There remain many issues to be addressed by the epidemiology community (see figure). Some of these issues involve challenges so great that we have perhaps not even begun to think about how we might start tackling them. We encourage authors to continue submitting articles on the theme of *Epidemiology in conflict*, so that we may be kept informed of developments in the field and promote the public health perspective in discussions about future conflicts.

**Figure:**
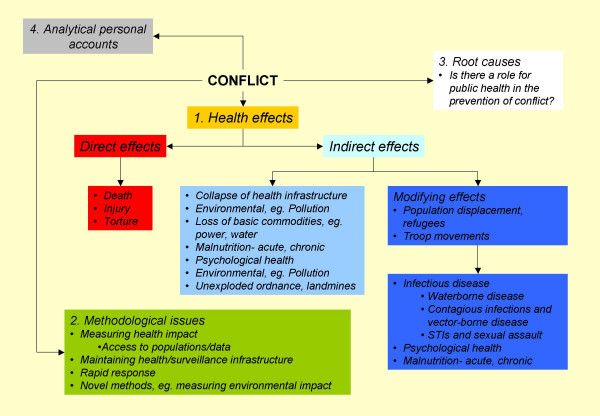
Conceptual framework for *Epidemiology in conflict*

We hope readers intending to take up these challenges will be informed, inspired and provoked into action. This is a call to arms, not against the barriers of physical inactivity and excessive caloric intake, the adaptability of infectious agents or the subtleties of gene-environment interactions, but against the sheer brutality of human beings killing each other. We encourage readers to research and discuss the humanitarian and public health consequences of this social disease. The knowledge gained will allow us to better mitigate the public health impacts of war, and place the public health community in a more informed position in discussions about possible interventions in future conflicts. The pen may yet prove to be mightier than the sword, but only as long as it keeps writing.
